# Immune Responses to Plasma-Derived Versus Recombinant FVIII Products

**DOI:** 10.3389/fimmu.2020.591878

**Published:** 2021-01-22

**Authors:** Flora Peyvandi, Syna Miri, Isabella Garagiola

**Affiliations:** ^1^Angelo Bianchi Bonomi Hemophilia and Thrombosis Center and Fondazione Luigi Villa, Fondazione IRCCS Ca' Granda Ospedale Maggiore Policlinico, Milan, Italy; ^2^Department of Pathophysiology and Transplantation, Università degli Studi di Milano, Milan, Italy; ^3^Department of Biomedical Sciences for Health, Università degli Studi di Milano, Milan, Italy

**Keywords:** recombinant products, plasma-derived products, inhibitors, von Willebrand factor, post-translational modification, cell lines

## Abstract

The most severe side effect of hemophilia treatment is the inhibitor development occurring in 30% of patients, during the earliest stages of treatment with factor (F)VIII concentrates. These catastrophic immune responses rapidly inactivate the infused FVIII, rendering the treatment ineffective. This complication is associated with a substantial morbidity and mortality. The risk factors involved in the onset of the inhibitors are both genetic and environmental. The source of FVIII products, i.e. plasma-derived or recombinant FVIII products, is considered one of the most relevant factors for inhibitor development. Numerous studies in the literature report conflicting data on the different immunogenicity of the products. The SIPPET randomized trial showed an increased in the inhibitor rate in patients using recombinant FVIII products than those receiving plasma-derived products in the first exposure days. The SIPPET randomized trial showed an increase in the inhibitor rate in patients using recombinant FVIII products compared to those treated with plasma-derived products in the first days of exposure. The potential increase in the immunogenicity of recombinant products can be attributed to several factors such as: the different post-translational modification in different cell lines, the presence of protein aggregates, and the role played by the chaperon protein of FVIII, the von Willebrand factor, which modulates the uptake of FVIII by antigen presenting cells (APCs). Furthermore, the presence of non-neutralizing antibodies against FVIII has shown to be in increased inhibitor development as demonstrated in a sub-analysis of the SIPPET study. In addition, the presence of the specific subclasses of the immunoglobulins may also be an important biomarker to indicate whether the inhibitor will evolve into a persistent neutralizing antibody or a transient one that would disappear without any specific treatment. Recently, the availability of novel non-replacement therapies as well as emicizumab, administered by weekly subcutaneous infusion, have significantly changed the quality of life of patients with inhibitors showing a considerable reduction of the annual bleeding rate and in most patients the absence of bleeding. Although, these novel drugs improve patients' quality of life, they do not abolish the need to infuse FVIII during acute bleeding or surgery. Therefore, the issue of immunogenicity against FVIII still remains an important side effect of hemophilia treatment.

## Introduction

Hemophilia A, an X-linked condition, is one of the most severe hereditary bleeding disorders caused by the deficiency of the coagulation factor VIII (FVIII) (1). Patients with severe hemophilia A (FVIII coagulant activity <0.01 IU/ml) suffer from repeated and spontaneous bleeding episodes mainly within muscles and joints, resulting in disabling musculoskeletal damage and chronic arthropathy (1). Prophylaxis has proven to be the elective treatment for the management of hemorrhagic events or to prevent joint damage, as demonstrated in young boys with severe hemophilia.

The main therapeutic strategy in hemophilia is the intravenous infusion of the deficient clotting factor to achieve appropriate hemostasis. Treatment is given in response to an acute bleeding episode (on-demand) or as long-term prophylaxis by infusion two to three times per week to prevent hemorrhages (2). Current treatment options, either plasma-derived or recombinant FVIII products, are effective in stopping and preventing hemorrhage, however, infusions of the therapeutic FVIII proteins in the first 50 exposure days (EDs) could lead to an undesired immune response, the development of antibodies against FVIII, called inhibitors. The appearance of inhibitors in hemophilia A patients should be seen as a natural immune system response to a non-self protein. The incidence of alloantibodies in the overall population with hemophilia A is estimated to be approximately 25% to 30% (3). Patients with severe hemophilia A are more prone to develop inhibitory antibodies than in patients with mild or moderate disease. Previously untreated patients (PUPs), which are patients unexposed to FVIII, are at greatest risk of inhibitor development within the first 10 to 20 EDs to therapeutically administered FVIII (4–6). Coagulation factor inhibitors may be neutralizing antibodies that lead to inactivation of the infused factor and non-neutralizing (i.e. non-inhibitory) antibodies that target non-functional epitopes on FVIII. Recently, the introduction of new non-replacement therapies (7) in routine clinical care seems to have solved the problem of treating patients with and without inhibitors. These drugs have demonstrated good effectiveness in the management of patients with inhibitors, significantly reducing the annual bleeding rate and resulting in numerous patients that remain bleed free. However, this kind of therapy only postpones the problem of inhibitor development in PUPs due to the need of FVIII infusions during bleeding events, trauma or surgery.

The generation of a neutralizing antibody might impact the efficacy of products resulting in a partial or complete abolishment of the replacement therapy, keeping patients vulnerable to bleeding symptoms and raising the risk of morbidity and mortality.

An explanation of this unwanted immune reaction could be the interaction between a large number of genetic and environmental risk factors involved in the process of anti-FVIII antibodies development (8). The source of FVIII products is still one of the most important and debated environmental risk factors implicated in inhibitor development, although the SIPPET (Study on Inhibitors in Plasma-Product Exposed Toddlers) randomized clinical trial has provided evidence of a higher risk of immunogenicity associated with recombinant FVIII products in PUPs (9). This potential increase of immunogenicity has some plausible biological explanations such as the different post-translational modifications (e.g. glycosylation and sulphation) caused by different cell lines during the manufacturing process and the protective role played by von Willebrand factor (VWF).

In addition to neutralizing antibodies, another important issue is the development of non-neutralizing antibodies, not only after the exposure to FVIII products but also before any treatment. The evaluation of immunoglobulin G (IgG) subclasses, before, during the first 50 EDs to FVIII and even six months after the development of inhibitors, could provide an essential information on how patients exposed to FVIII could develop transient or persistent anti-FVIII neutralizing antibodies.

This review article reports data available in the literature on how the immune response may vary depending on the type of FVIII product used.

## Differential Immunogenicity Between the Classes of FVIII Products

### Observational and Randomized Studies

The manufacturing process of plasma-derived FVIII products has been subject of widespread disagreement and controversy on the risk of inhibitor development. In particular, viral inactivation steps (e.g. pasteurization and solvent-detergent treatment) probably render plasma-derived products more immunogenic (10). Since the introduction of recombinant FVIII, these products have raised concern on their higher immunogenicity than plasma-derived products (11).

A range of observational studies have sought to evaluate any differential risk of inhibitor development between the classes of plasma-derived and recombinant FVIII products, and also between the different labels of recombinant products over the years (12–15). These studies have yielded different results and suffer from the limitations of observational studies, such as heterogeneity in study design, confounding by indication and in particular from possible selection bias. Furthermore, over time there have been changes in the manufacturing process of each single product and changes in treatment regimens between different centers, hence comparison between products is not always possible. These factors have introduced a challenge in the interpretation of results of such studies. The CANAL and RODIN studies in large cohorts of PUPs with severe hemophilia A (13, 14) found no significant difference in the risk of inhibitor development between plasma-derived and recombinant products. Additional information was achieved by the RODIN study demonstrating a divergent immune response between different recombinant products (14). A higher incidence of inhibitors has been provided in patients who were treated with second-generation full-length recombinant products produced in baby hamster kidney (BHK) cells than those treated with third-generation products produced in Chinese hamster ovary (CHO) cells. These results were then confirmed in other additional studies conducted in French and UK cohorts of PUPs with severe hemophilia A (15, 16). However, to solve definitively the inhibitor development puzzle about FVIII source, the scientific community asserted the need to carry out a randomized clinical trial (17).

Randomized controlled trials are the basis of evidence-based medicine as they supply the highest level of information and recommendations for therapeutic options. Treatment for hemophilia is founded on very few randomized controlled trials, partly because of the relative rarity of the disease and ethical aspects of randomization but also because of the excellent relationship between plasma levels of FVIII and clinical outcomes. Notwithstanding the expected difficulties in designing and conducting a randomized controlled trial in hemophilia, the SIPPET study was initiated in 2009, published in 2017 providing definitive answers regarding the different immunogenicity between recombinant and plasma-derived FVIII products (9). The results rising from SIPPET showed a higher risk of developing inhibitors in patients treated with recombinant FVIII products (87%) than those treated with plasma-derived FVIII products.

Subsequently, a European Hemophilia Safety Surveillance Project (EUHASS) and EMA, after reviewing SIPPET data, concluded that clear evidence in the rate of development of inhibitors between plasma-derived and recombinant products had not been demonstrated (18, 19). This study was criticized for the geographically unusual study population which had a higher representation of some ethnic groups (Egypt, India and Iran). Other issues were related to the follow-up of up to 50 days of exposure, and the choice of a lower than usual inhibitory titer threshold (0.4 BU). All these aspects have been critically addressed in a subsequent review article (20). In addition, these findings are clinically important, because the development of FVIII alloantibodies is currently the major therapeutic complication in hemophilia A, that causes a marked increase in morbidity, mortality and treatment costs.

Concordant findings with the SIPPET randomized trial were reported in a French national cohort study (16). This study compared inhibitor incidence among large groups of PUPs receiving single FVIII products, including one plasma-derived product and two recombinant FVIII products. A higher risk of inhibitor development was reported in patients treated with recombinant products, and the cumulative incidence of inhibitors was almost twice as high in PUPs treated with second-generation recombinant products as in those treated with plasma-derived. For high-titer inhibitors, the cumulative incidence at 75 EDs was 12.7% (95% CI: 7.7–20.6) with plasma-derived, 20.4% (95% CI: 14.0–29.1) with third generation recombinant product, and 31.6% (95% CI: 23.5–41.7) with second-generation recombinant product. For high-titer inhibitors, adjusted hazard ratio of third-generation *versus* plasma-derived was 1.64 (95% CI: 0.82–3.25). A similar result had been observed in the SIPPET study, in which the adjusted hazard ratio for recombinant FVIII versus plasma-derived FVIII was 1.69 (95% CI: 0.96–2.98). The same trend was observed for second-generation recombinant product *versus* plasma-derived, adjusted hazard ratio was 2.81 (95% CI: 1.44–5.49).

New emerging products have been introduced in the last 4 to 5 years, including rVIII-SingleChain. This novel recombinant FVIII product is a B domain deleted recombinant FVIII with an intrinsic stability of the FVIII molecule which reduces the potential dissociation of the heavy and light chains of FVIII increasing its affinity to von Willebrand factor (21). rVIII-SingleChain is expressed in CHO cells and no human- or animal-derived proteins are added in the production steps or in the formulation stages. Interim analysis of the phase III extension study has been proposed to evaluate the safety and efficacy of rVIII-SingleChain in PUPs and recently the results have been presented during the American Society of Hematology (ASH) 2019 annual meeting (22). Twenty-three PUPs were treated with rVIII-SingleChain and assigned by the investigator to a prophylaxis or on-demand treatment regimen. Twelve subjects had positive inhibitor titer (52%, 95% CI: 31–73); six PUPs (26%) developed a high-titer (peak titer ≥5 BU/ml), and six (26%) low-titer inhibitors. (peak titer <5 BU/ml). The median EDs for inhibitor development was 10 (range, 4–23).

For almost all recently approved extended half-life products for hemophilia A and B, there is still no information on inhibitor development in PUPs except for extended half-life products Fc-fused. Despite previous studies on mice in favor of a protective effect of the Fc fragment in rFVIII-Fc (23, 24), preliminary clinical trial results showed an overall inhibitor development of 27.7% (95% CI: 19.3–37.5) using rFVIII-Fc, equivalent to standard products (25).

### Genetic Risk Factors for Inhibitor Development

Genetic factors, in particular the *F8* gene mutations, are strongly related to inhibitor development. Mainly null mutations, such as nonsense mutations and large deletions, seem to be associated to the highest risk of developing inhibitors (26). The involvement of immune response genes (e.g. the human leukocyte antigen complex) and proteins (e.g. cytokines) in modulating the risk of inhibitor development has been studied with controversial results on their role. In addition, ethnicity also plays a role in the development of inhibitors (27). African-Americans and Latinos with hemophilia A have higher inhibitor risk than Caucasians with prevalence of inhibitors in Black patients twice higher than White patients (28, 29).

A recent publication has examined whether the type of *F8* gene mutation may have an effect on the development of the inhibitor by considering the type of product used for treatment (30). This study found that a low risk of inhibitor development was observed for patients with low genetic risk (missense mutation) and treated with plasma-derived FVIII, whereas patients with a high genetic risk profile (intron 22 inversion, intron 1 inversion, frameshift, nonsense, large deletion) and treated with recombinant FVIII have a significantly higher risk of inhibitor development.

### Subanalysis Within the SIPPET Trial

#### Different Timing of Inhibitor Development in Recombinant Versus Plasma-Derived FVIII Concentrates

The topic on the time course of inhibitor development has never been extensively analyzed. In the literature, there are few data on the exact time of inhibitor development and mainly it has not been clarified whether there is a difference in the risk of inhibitor development between the two classes of products over time. Data from the first study treating this question dates back to 1994, where the authors analyzed the risk of inhibitor development in patients treated with full-length recombinant FVIII, and reported that the median number of EDs for the patients who had developed inhibitors was 9 EDs (range 3 to 45) (31). These findings were then verified by two independent studies, confirming that the median EDs in which inhibitor developed was 9 in patients treated with full-length recombinant FVIII in both studies (32, 33). A subsequent study analyzed the inhibitor occurrence only in PUPs or minimally treated patients (MTPs) after exposure to a plasma-derived product. In this case, seven out of 99 patients developed inhibitors (7.1%, 95% CI: 3–14) after a median EDs of 11 (range 4–22) (34). In the CANAL and RODIN studies, in which PUPs were treated with plasma-derived or recombinant FVIII products, inhibitory antibodies developed after a median of 14 (range, 8–21) and 15 EDs (IQR: 10 to 20) respectively without a significant difference between the two products (13, 14). Three studies recorded more or less the same time of inhibitor development (18, 35, 36). A more precise assessment of the timing of inhibitor occurrence became available from the SIPPET study (6). The study envisaged inhibitor titer monitoring at strict and frequent time intervals, usually every 5 EDs in patients treated with different types of FVIII products. This stringency in inhibitor testing allowed to establish with a higher precision the time course of inhibitor occurrence. The highest rate of inhibitors developed in the first 10 EDs, with a large variation between recombinant and plasma-derived FVIII during the first 5 EDs (6). Patients treated with recombinant products were found to have a three- to four-fold higher risk of inhibitor development, including high-titer inhibitors (**Table 1**), when compared to patients treated with plasma-derived FVIII during the first five EDs. Plasma-derived products seemed to have a belated immunogenicity. Different mechanisms could play a role in such a rapid reaction to recombinant products. It is biologically feasible that more post-translational modifications (e.g. glycosylation) raise with plasma-derived FVIII than with recombinant FVIII. The fraction of free FVIII is unable to bind von Willebrand factor (VWF), masking FVIII recognition. Furthermore, plasma-derived products may contain immunomodulating human proteins which may play also a role in inducing tolerance. Further basic research studies are needed to confirm such speculations.

**Table 1:** Risk of inhibitor incidence over time.

ED	Plasma-derived FVIII			Recombinant FVIII		
	Number of treated patients	Number of patients with inhibitors	Incidence %(95% CI)	Number of treated patients	Number of patients with inhibitors	Incidence %(95% CI)
**(A) All inhibitors**						
0–5	125	4	3.2 (1.3–7.9)	126	12	9.5 (5.5–15.9)
6–10	112	15	13.4 (8.3–20.9)	103	19	18.4 (12.1–27.0)
11–15	92	3	3.3 (1.1–9.2)	76	6	7.9 (3.7–16.2)
16–20	84	4	4.8 (1.9–11.6)	65	5	7.7 (3.3–16.8)
21–25	76	0	0.0 (0–4.8)	56	2	3.6 (1.0–12.1)
26–30	65	2	3.1 (0.8–10.5)	52	0	0.0 (0–6.9)
**(B) High-titer inhibitors**						
0–5	125	3	2.4 (0.8–6.8)	126	12	9.5 (5.5–15.9)
6–10	112	12	10.7 (6.2–17.8)	103	11	10.7 (6.1–18.1)
11–15	92	2	2.2 (0.6–7.6)	76	4	5.3 (2.1–12.8)
16–20	84	2	2.4 (0.7–8.3)	65	2	3.1 (0.8–10.5)
21–25	76	0	0.0 (0–4.8)	56	1	1.8 (0.3–9.4)
26–30	65	0	0.0 (0–5.6)	52	0	0.0 (0–6.9)

(A) The risk of all inhibitors based on to the class of FVIII products (recombinant or plasma-derived). (B) The risk of high-titer inhibitors. This table was adapted from Table 1 in Peyvandi et al. J Thromb Haemost 2018 (6).

#### Total Anti-FVIII Antibodies and IgG Subclasses

Anti-FVIII antibodies in patients with hemophilia A comprise both neutralizing (inhibitors) and non-neutralizing antibodies. Studies in other diseases demonstrated that non-neutralizing antibodies directed against therapeutic proteins may influence their pharmacokinetic and pharmacodynamic profiles. The non-neutralizing anti-FVIII antibodies have been detected not only in hemophilia patients but also in healthy individuals (37).

**Figure 1:**
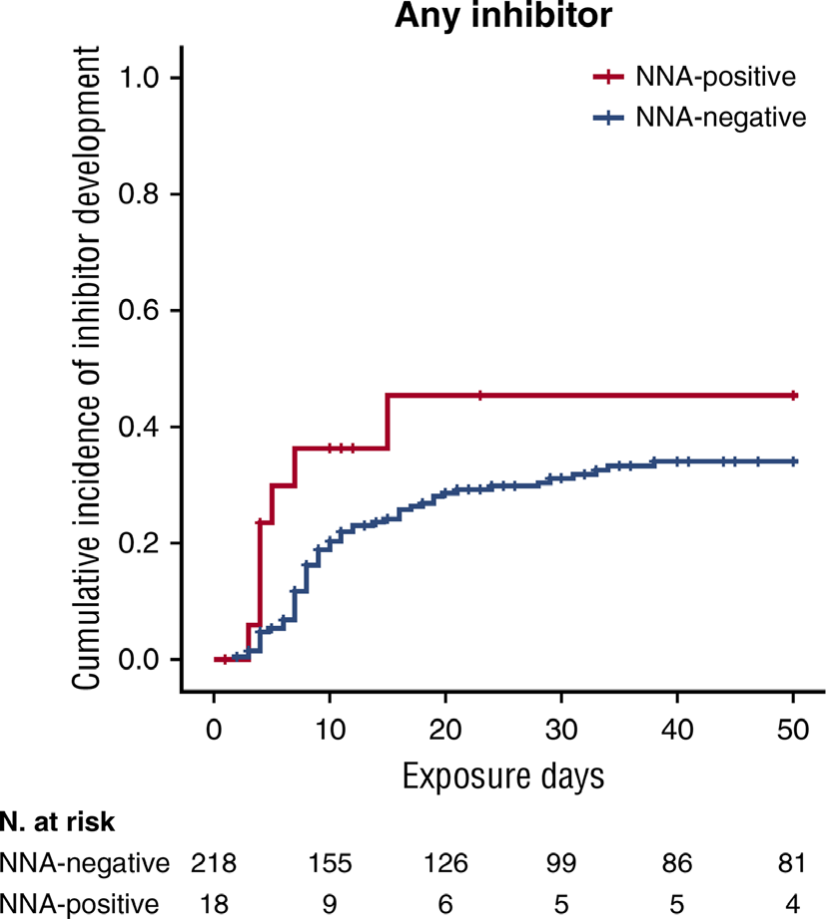
Kaplan-Meier survival curves for inhibitor development based on NNA presence. Cumulative incidence for all inhibitors. This figure was adapted from Figure 1 (A) in Cannavò et al. Blood 2017 (28).

Recently, the involvement of non-neutralizing antibody in inhibitor development has been investigated in a relevant subpopulation of the SIPPET cohort. Subjects enrolled were PUPs or MTPs with blood components, randomly assigned to receive either plasma-derived or recombinant FVIII products. Serial plasma samples, scheduled at fixed time points, from the screening visit up to conclusion were planned in the study protocol (38). The use of stored biological samples for secondary end points has been stated in the protocol and informed consent form, these serial plasma samples available were used to detect the presence of non-neutralizing antibodies. A cumulative incidence of 45.4% (95% CI: 19.5–71.3) was observed among patients who were positive for non-neutralizing antibodies at screening and subsequently developed an inhibitor, while patients negative for non-neutralizing antibodies at screening showed a cumulative incidence of 34.0% (95% CI, 27.1–40.9). This study demonstrated that the presence of non-neutralizing antibodies at screening was associated with an increased probability of inhibitor development of 83% (**Figure 1**), while the incidence was almost tripled for high-titer inhibitors (38). Therefore, the presence of non-neutralizing antibodies may be considered an additional marker predisposing to inhibitor development.

Antibodies directed against FVIII consist of a polyclonal IgG population. In human, four IgG subclasses exist and differ greatly in function, particularly in relation to complement activation and engagement with cellular Fc receptor (FcγR) binding (39). Immunoglobulins are produced by B cells only after they have undergone an antigen-driven differentiation, during which follicular B cells turn into short-lived plasma blasts which secrete antibodies that are mainly of the IgM and IgG isotypes (40). Sequential switching of the subclass of immunoglobulins is linked to higher levels of somatic hypermutation of their variable region (41).

Studies executed in the 1990s, reported that all IgG subclasses with a majority of IgG1 and IgG4 were involved in the immune response to FVIII in patients with hemophilia A (42). A more recent study evaluated the prevalence of IgG subclasses of anti-FVIII antibodies in four groups of individuals: healthy subjects, hemophilia A patients with and without inhibitors, and acquired hemophilia A patients (43). Significant differences between IgG subclasses of anti-FVIII antibodies within the different study groups were found. IgG1 and IgG4 were the most substantial IgG subclasses found in patients with inhibitors and in patients with acquired hemophilia. Instead, IgG2 and IgG3 subclasses were less detected in these two groups of patients. The subclasses IgG3 and IgG1 emerged as dominant subclasses in the healthy group, in which IgG4 was completely absent. The same situation was detected in patients without FVIII inhibitors. The most interesting finding of this study was the detection of IgG4 subclasses exclusively in hemophilia A patients with inhibitors. In a subsequent study (44), IgG1 and IgG4 exclusively characterized hemophilia A patients with persistent inhibitors and in acquired hemophilia A patients, confirming the previous data. Furthermore, studies on autoimmune diseases, particularly type 1 diabetes mellitus, reported the importance of the affinity of antibodies as a potential biomarker for eventually developing the disease. Consistently, the affinity of FVIII-specific antibodies, split into isotypes and IgG subclasses, has been evaluated (44). The affinity of FVIII-specific antibodies was higher in patients with persistent inhibitors compared to the affinity in patients without inhibitors and healthy individuals. In particular, FVIII-specific IgG4 in patients with inhibitors expressed the highest affinity compared to all other IgG subclasses (44). Therefore, the presence of high-affinity FVIII-specific IgG4 might be considered as a biomarker for the development of inhibitors. The authors assumed that the higher affinity FVIII antibodies in inhibitor patients are produced by plasma cells differentiated from follicular pathways in germinal centers and then migrate to the bone marrow. Instead, the lower affinity antibodies in patients without inhibitors and in healthy individuals are more likely generated by plasma cells deriving from extrafollicular pathways or from marginal-zone B cells.

Boylan et al. (45) used an immunofluorescence immune assay to detect all anti-FVIII antibodies in patients with hemophilia A with and without inhibitors. Evaluation of antibody profiles indicated that the presence of anti-FVIII IgG1, IgG2 or IgG4 was qualitatively and quantitatively related to the presence of a FVIII inhibitor.

In addition, the antibody subclass profiles have been monitored in serial sampling of hemophilia A patients to identify a specific IgG as a predictive marker for inhibitor development. The preliminary data showed that patients with anti-FVIII IgG1 were most likely to develop inhibitors and this subclass may be considered as an early biomarker for inhibitor development (45).

In the human inhibitor PUP study (HIPS), distinct subclasses of IgG were identified in distinct groups of patients (46). The group of patients with FVIII inhibitors expressed firstly high-affinity IgG1 followed by high-affinity IgG3 and then IgG4. In the group of patients with only non-neutralizing antibodies, IgG1 antibodies were detected and no other IgG subclass. Another group represented by patients with low-titer inhibitors, of which one with transient FVIII inhibitors, developed only high-affinity IgG1. These data partially reflect those reported in another study on patients with multiple sclerosis in which treatment with interferon-*β* 1b, a recombinant product, induces the production of neutralizing and non-neutralizing antibodies (47). The immunologic profile of the IgG subclasses in patients with neutralizing antibodies was represented by IgG2 and IgG4, and there was a strong correlation between IgG4 and the titer of neutralizing antibodies. Contrarily, patients with non-neutralizing antibodies expressed IgG1 antibodies. The same study group in a subsequent analysis reported that patients with or without transient neutralizing antibodies displayed predominantly IgG1 and IgG3 subclasses, lower antibody titers and antibody binding affinity compared to patients with persistent neutralizing antibodies, in whom the most frequent IgG subclasses were IgG2 and IgG4 (48). The limit of these two studies was the low number of analyzed patients.

Recently, a subanalysis of the SIPPET trial investigated the predictive value of IgG subclasses and the risk to develop a persistent or transient type of inhibitor (49). The concomitant presence of more than one IgG subclass and high-titer inhibitor was associated with a high risk to develop a persistent inhibitor.

A temporal model proposed on the dynamics of isotype switching has been proposed by Collins et al. in the context of pathogen infections (50). IgM^+^ B cells switch to both IgE and IgG3 early in the germinal center. Subsequently, IgG1 cells emerge, followed by IgG2 cells and finally, if antigens persist, by IgG4-producing cells. The IgG3 response occurs early, and their nature is relatively transient and of low affinity (51). The relatively early appearance of IgG1 in the immune response could lead to premature antigen clearance, preventing the appearance of IgG2 and IgG4 antibodies. Shortly after switch to IgG1, IgG2 response emerges from germinal center reaction. In certain circumstances, quick switching leads to a response that is dominated by IgG2. Indeed, a conspicuous part of the antibody response to many protein antigens is often dominated by IgG2 (52). IgG4 cells are the last to appear from the germinal center reaction and are therefore likely to be the highest affinity antibodies. This temporal model configures sequential class switching during a first, persisting exposure to antigen. The nature of isotype expression in a recall response will result from the ability of class-switched cells to differentiate into memory cells during the primary response.

With this background, it remains to be understood which anti-FVIII IgG subclasses could be a relevant predictive marker for inhibitor development, and which IgG subclasses could lead patients to have endogenous tolerance preventing the formation of persisting neutralizing antibodies which require a specific therapeutic approach.

## Post-Translational Modifications and Cell Types for Manufacturing of Recombinant FVIII Products

The chemical changes of proteins after translation are referred to as post-translational modifications. The formation of disulfide bonds, or covalent addition or removal of low-molecular-weight groups, are the most frequent modifications, thus leading to acetylation, amidation, biotinylation, glycation (nonenzymatic conjugation with carbohydrates), glycosylation (enzymatic conjugation with carbohydrates), hydroxylation, methylation, etc. An important role is played by post-translational modifications in regulating the folding of proteins, their targeting to specific subcellular compartments, their interaction with ligands or other proteins, their functional state, as well as their immunogenicity.

Glycosylation is a complex process that serves to expand the diversity of the proteome and is of critical importance especially for the synthesis of recombinant proteins. Glycosylation involves the addition to proteins of a diverse option of sugar moiety varying from simple monosaccharide modifications to highly complex branched polysaccharides. “Asparagine-linked (*N*-linked) or serine/threonine-linked (*O*-linked) oligosaccharides are major structural components of many cell surface and secreted proteins” (53). The glycosylation profile changes substantially depending on the cell type used for the manufacturing of recombinant proteins. The expression systems of choice to produce most therapeutic recombinant plasma proteins, able to perform complex post-translational modifications are mammalian host cells, as Chinese Hamster Ovary (CHO) cells and Baby Hamster Kidney (BHK) cells. CHO cells have been widely used in laboratories since 1919 for large-scale commercial production of recombinant proteins. Many companies have also been successfully producing several recombinant FVIII products in CHO cells, whereas BHK cells were used only by one company to produce a recombinant FVIII product (54).

To date, recombinant FVIII is the largest and most complex protein manufactured by recombinant DNA technology. FVIII is a multi-domain heterodimer that comprises 2332 amino acids assembled into six structural domains, organized in a heavy chain (A1-A2-B domains) and a light chain (A3-C1-C2 domains). FVIII is heavily glycosylated, *N*- and *O*-glycosylation, and carries sulphated tyrosine residues (55).

The B domain is encoded entirely by a single large exon 14, and represents the largest domain in FVIII, is abundantly glycosylated and highly preserved, with 907 residues making up 40% of the entire sequence (56, 57). This domain is relatively dispensable for procoagulant activity (57, 58). Its high degree of glycosylation consists of 19 asparagine *N*-linked glycosylation attachment sites (Asn-X-Thr/Ser) and at least 7 *O*-linked glycans present in the FVIII protein. This region may be significant for intracellular processing and trafficking during protein synthesis (59). The other 6 asparagine *N*-linked glycosylation sites found outside B domain are located at 41 (A1), 239 (A1), 582 (A2), 1685 (a3), 1810 (A3) and 2118 (C1) residues (60). Glycosylation is considered one of the most important and conditioning processes influencing the biological activity, serum half-life and immunogenicity of FVIII. Already in 1992, it was observed that glycosylation profiles differed between plasma-derived and recombinant FVIII products (61). The major difference in the sugar chains of plasma-derived FVIII compared to recombinant FVIII was that some of the outer chains of the complex-type sugar chains of recombinant FVIII contain the Galα1-3Gal group. Detection of this group in the sugar chains of recombinant FVIII is not surprising, since most mammalian cells contain the α-1,3-galactosyltransferase responsible for the addition of the Galα1-3Gal group to the glycoproteins. Moreover, in the same study, variations in the glycosylation pattern between different recombinant FVIII products, produced in different cell lines, have also been described (61). The increased immunogenicity of recombinant FVIII products has been attributed to deglycosylation or incomplete *N*-linked glycosylation exposing previously polysaccharide-protected epitopes (62). This data was also demonstrated in animal models (63, 64) and subsequently in separate observational studies (12, 14, 16). Recently, glycosylation profiles of second-generation BHK recombinant FVIII and third-generation CHO recombinant FVIII have been characterized by performing a glycopeptide analysis by liquid chromatography and mass spectrometry. In addition, their role in the development of inhibitors in hemophilia A mice has been evaluated (65). This study confirmed the data previously reported that *N*-linked glycans shield epitopes of FVIII protein and the authors concluded that the increased immunogenicity of second-generation BHK recombinant FVIII is, in part, correlated to incomplete *N*-linked glycosylation that exhibit FVIII epitopes to IgM and IgG, that may promote the formation of immune complexes.

The B domain is dispensable for the procoagulant activity of FVIII and it is removed upon cleavage and activation by thrombin (55). The key role played by the B domain is in the FVIII secretion pathway (59). Absence of the B domain results in a reduction of FVIII secretion, although it still occurs. A recombinant FVIII product without B domain has been produced, although the B domain has not been fully deleted and a small portion is retained (66) in order to facilitate its secretion. Different levels of immunogenicity were reported (9, 13, 14), indeed prospective meta-analysis studies showed an increased risk of inhibitor formation compared to full-length recombinant products in previously treated patients (67–69). A comparison between the B domain deleted product and two full-length recombinant products have been performed in both *in vitro* and *in vivo* studies (70). This study observed that the endocytosis of the B domain deleted product by monocyte-derived dendritic cells (MO-DCs) was lower or equal to full-length products. Furthermore, the inhibitor levels induced by B domain deleted products in deficient mice were comparable to that of full-length products (70).

The higher immunogenicity of recombinants FVIII products was also associated with the presence of aggregates particularly in recombinant product formulations. Under certain conditions, recombinant FVIII has a tendency to aggregate, and this propensity appears to be most often associated with structural changes in the molecule attributed to post-translational modifications and essentially to differential glycosylation profile. The removal of sugar chains from recombinant FVIII products has been shown to determine their partial aggregation, in particular the removal of N-glycan at positions N-1283 and N-2131 respectively in the A3 and C1 domains of FVIII. This deglycosylation leads to a change in the conformational structure of the light chain resulting in protein aggregation, as suggested in Kosloski's study (64). Moreover, in the last few years, the presence of aggregates has been found in different pharmaceutical formulations of recombinant FVIII products, notably in second-generation BHK and third-generation CHO recombinant FVIII, by the use of sedimentation velocity analytical ultracentrifugation (71). Furthermore, larger aggregates size was found in the second-generation recombinant FVIII compared to third-generation recombinant products (71), with knowledge that the immunogenicity of these protein aggregates is related to their size increase, as demonstrated in some experimental systems (72). The presence of large aggregates has been recently confirmed using dynamic light scattering spectroscopy (73). The increase in immunogenicity, proportional with the presence of (large) aggregates is not well understood. However, some studies have specified that aggregates can promote an increase in antibody titers that may be due to both B cell and T cell responses associated with an increase in the secretion of proinflammatory cytokines IL-6 and IL-17 (74).

An additional post-translational modification, which may have an impact on immunogenicity, includes sulphation of specific tyrosine residues of the recombinant proteins. There are six sulphated tyrosines in the human FVIII, four in the heavy chain and two and in the light chain. Several studies suggested that sulphation of the specific amino acid residue Tyr1680 (Tyr1699 in HGVS nomenclature) is crucial for the capacity of FVIII to bind VWF and consequently plays a pivotal role in its stability (75). Interaction with endogenous VWF is suggested to shield circulating FVIII molecules by decreasing the uptake of unbound FVIII from endocytosis by dendritic cells, lowering effector cell presentation and decreasing immunogenic potential. Experiments performed in animal model showed higher levels of anti-FVIII in mice treated with recombinant FVIII compared to plasma-derived concentrates (63). A following study proved that Tyr1680 is not completely sulphated in recombinant FVIII products compared to plasma-derived. These findings suggest two potential impacts of sulphation on FVIII immunogenicity. On the one hand, the unbound portion of recombinant FVIII molecules could be more immunogenic than the VWF-bound recombinant FVIII. On the other hand, the sulphated form of recombinant proteins has a changed structural conformation that facilitates antigen presenting cell uptake (76).

## Role of VWF

The protective role played by VWF in modulating FVIII immunogenicity has been deeply investigated. The amount of VWF is variable in the different types of FVIII products derived from plasma, depending on the purification process, in contrast to the recombinant FVIII concentrates which are fully free of VWF.

Under physiologic conditions, VWF binds to FVIII after its release in the circulation and acts as protector and chaperone molecule for the procoagulant factor. VWF protects FVIII from premature inactivation and clearance from the circulation, preserves the FVIII heterodimeric structure, regulates its activation by thrombin and further modulates its removal by lipoprotein-related receptors.

To assess the relative variability in inhibitor development between different FVIII products with non-equivalent content of VWF, studies were conducted in FVIII knockout mice. The findings obtained from this first systematic comparison on the relative immunogenicity of FVIII products showed that an increased risk of anti-FVIII inhibitor is associated to FVIII concentrates with no (recombinant products) or reduced amount of VWF (plasma-derived). Further addition of VWF to plasma-derived and recombinant FVIII products resulted in a significant decrease (p<0.05) of inhibitor titer (77). A confirmation of these data has been successfully obtained in FVIII deficient mice in which the anti-FVIII IgG titers were 2.4- to 3.2-fold higher in mice treated with recombinant FVIII concentrates than those treated with plasma-derived (p<0.055). However, the administration of plasma-derived alone induced measurable levels of anti-FVIII IgG, indicating that a large molar excess of VWF reduced the immunogenicity of FVIII but did not completely suppress FVIII immunogenicity (63). Protective effect of VWF on the immunogenicity of the FVIII has been further investigated in injected mice with recombinant FVIII pre-incubated with VWF showing a significant reduction in the anti-FVIII IgG levels (p<0.0001) relatively comparable to the levels obtained with the plasma-derived products (63). *In vitro* experiments, using human dendritic cells (DCs) generated from circulating monocytes (Mo-DCs) of healthy blood donors, have highlighted that VWF behaves like an immunoprotective chaperone for FVIII by preventing endocytosis by DCs and subsequent presentation to FVIII-specific T cells. Furthermore, the VWF preserves FVIII in a dose-dependent manner from being endocytosed by DCs (78). A consequence of the reduced internalization of FVIII is a decrease in the capacity to activate a FVIII-specific CD4+ T cells clone, thus demonstrating that smaller amounts of FVIII have been processed and presented to T cells. Using Mo-DCs, a potent endocytic receptor [C-type lectin receptors (CD206)] for mannose-ending glycans expressed on the heavy and light chain of FVIII has been identified. VWF interfered in the interaction between FVIII with lectin receptor, suggesting that the intrinsic mannose-dependent immunogenicity of FVIII is abolished by endogenous immunochaperones (79).

A reduction in FVIII inhibitor development after treatment of hemophilia A mice with plasma-derived has been reproduced by Qadura et al. (80), however this study failed to confirm the reduction in FVIII inhibitor levels when FVIII products were pre-incubated with VWF. Moreover, a different profile of immune gene expression in splenic DCs, and also differences in the secondary immune response after plasma-derived FVIII infusion *versus* recombinant FVIII administration in hemophilia A mice have been proved. Notwithstanding, administration of recombinant FVIII induced the release of T helper 1 (Th1) cell cytokines, whereas plasma-derived induced the release of Th2 cytokines (80). Reding et al. have hinted a more important role of Th1 cells in the immune response to FVIII in the long-term maintenance of anti-FVIII antibody synthesis (81).

The immunoprotective role of VWF toward FVIII was explored *in vivo*, using bone marrow-derived DCs (82). Preincubation of FVIII with VWF reduced the endocytosis of FVIII by murine bone marrow-derived DCs in a dose dependent manner. In addition, a large molar excess of VWF reduced the immunogenicity of FVIII in the murine model but failed to completely abolish FVIII immunogenicity. Surprisingly, the presence of VWF increased the amount of FVIII accumulated in the marginal zone of the spleen. The marginal zone B cells play an important role in determining tolerance to exogenous FVIII in the mouse model. To sum up this study, VWF may have at least two roles in FVIII immunogenicity: VWF may reduce the endocytosis of exogenous FVIII by antigen-presenting cells (APCs), through the prevention of interaction between FVIII and an unknown endocytic receptor. In addition, VWF may allow an increased processing of FVIII by B cells in the marginal zone of the spleen, thus promoting the development of regulatory immune processes that in turn mitigate the magnitude of the anti-FVIII immune response.

The modulatory role of VWF on internalization of FVIII by DCs has been shown by several additional studies (78, 83, 84). FVIII is rapidly internalized through C1 domain binding to an unidentified receptor in absence of VWF. When the FVIII/VWF complexes bind to APCs, FVIII dissociates from VWF to bind to an endocytic receptor, whereas VWF remains predominantly on the cell surface, without being internalized (85).

The internalization of FVIII/VWF complexes by APCs progresses in a differently way when compared to non-VWF bound FVIII. Several FVIII recombinant products showed an incomplete sulphation of Tyr1699 of FVIII, reducing VWF binding and leaving the amount of FVIII without a sulphated Tyr1699 to be internalized in a VWF-independent manner compared to other products with a normal sulphated Tyr1699, e.g. plasma-derived products or recombinant FVIII produced in HEK cells. In a recent study (86), a FVIII-nanobody fusion protein had a high binding affinity to VWF. The results showed that a stabilized FVIII/VWF complex, favored by nanobody fusion protein, was associated with a prolonged survival of FVIII and a reduced immune response against FVIII.

## Conclusion

Considerable advances in the manufacturing of hemophilia drugs in recent decades have guaranteed a major efficacy of products leading to a joint health preservation with prophylaxis, reduction in morbidity and mortality and the improvements of quality of life among hemophilic patients. Despite so, the development of inhibitors still remains one of the most relevant complications and the major challenge in the treatment of hemophilia. Inhibitor development is a multifactorial process, and the type of FVIII product is one of the main factors with a relevant influence on inhibitor formation. Current knowledge suggests that there are biological differences between plasma-derived and recombinant products, such as cell line selection, post-translational modifications, VWF content, and other properties, which could trigger a different immune response in several classes of FVIII products in PUPs. Providing a better understanding of the different mechanisms underlying the peculiar immunogenicity of these two classes of products is of extreme importance. The publication of the SIPPET study and its post-hoc analyses have influenced the clinical practice of hemophilia (87) resulting in difficulty in decision-making of when to start treatment in PUPs and with which product, even though in some countries recombinant concentrates are considered the treatment of choice due to their low probability of pathogen transmission (88). Currently available new extended half-life FVIII products in clinical practice have proven their efficacy, however for almost all these products, there is still no information on the rate of inhibitor development in PUPs except for extended half-life products Fc-fused, whose inhibitor development rate seem equivalent to standard products (24, 25).

Moreover, new non-replacement therapies (anti-TFPI antibody, bispecific antibody, siRNA targeting antithrombin and SerpinPC) are currently being evaluated for routine prophylaxis in patients with and without inhibitors and may overcome issues with adherence to prophylaxis even if they do not fully solve the inhibitor problem at the time of FVIII exposure particularly in PUPs. To date, clinical experience with the use of emicizumab for the treatment of PUPs is satisfactory, although the risk of inhibitor development has only been postponed but not solved.

In conclusion, considering the striking evolution in hemophilia treatment, the formation of inhibitors remains a serious problem in the treatment of patients. The need to clarify the pathophysiological aspects of inhibitor development, together with the manufacturing of products with reduced immunogenicity, will probably be the key issue in the coming years.

## Author Contributions

FP and IG contributed to the design and drafting of the manuscript. FP and IG revised the manuscript with the support of SM. All authors contributed to the article and approved the submitted version.

## Conflict of Interest

FP has received honoraria for participating as a speaker at satellite symposia organized by Grifols, Sanofi, Sobi, and Takeda. She also reports participation on the advisory boards of Sanofi and Sobi.

The remaining authors declare that the research was conducted in the absence of any commercial or financial relationships that could be construed as a potential conflict of interest.
